# Geochemical dispersion of gold-bearing quartz veins in the Wadi Abu Khusheiba area in Southern Jordan

**DOI:** 10.1186/s12932-024-00085-9

**Published:** 2024-03-02

**Authors:** Mariam Mosleh, Jasmi Hafiz Abdul Aziz, Muhammad Hatta Roselee, Ahmed Al-Shorman, Mahmoud Al Tamimi, Abdelkarim Alsoudi

**Affiliations:** 1 Department of Geology, Faculty of Science, Universiti Malaya, 50603 Kuala Lumpur, Malaysia; 2 Department of applied Earth and Environmental Sciences, Faculty of Earth and Environment Sciences - Al al-Bayt University, 130040, Mafraq, 25113 Jordan; 3 Department of Archaeology, Yarmouk University, 566, Irbid, 21163 Jordan; 4 Department of Earth and Environmental Sciences, Yarmouk University, 566, Irbid, 21163 Jordan; 5 Amman, Jordan

**Keywords:** Gold-bearing quartz vein, Hydrothermal geochemical dispersion, Aheimir volcanic suite, Wadi Abu Khusheiba, Jordan

## Abstract

This study delves into the geochemical dispersion of gold-bearing quartz veins in the Wadi Abu Khusheiba area, southern Jordan, with a focus on uncovering the complex patterns of mineralization and their geological significance. Employing an in-depth geochemical analysis of 24 rock samples from the region, we identified that these samples are predominantly hosted by oversaturated rhyolitic rocks, characterized by high SiO_2_ content and abundant free Quartz and orthoclase minerals. The mineralized zone of the quartz veins is particularly notable for its gold and silver concentrations, with maximum values reaching up to 5 ppm for gold and 18 ppm for silver. Our investigation into the elemental correlations revealed nuanced relationships, dependent on the 21 sample and analyzed at confidence level of (85%). Contrary to initial assumptions, we did not find a significant positive correlation between gold (Au) and arsenic (As), nor significant negative correlations between gold and other trace elements. These insights are critical for understanding the geochemical behavior of gold in the area and offer a nuanced view of elemental associations. The results of this study are significant for both academic research and practical exploration. They enhance our comprehension of the geological history and mineralization processes in Wadi Abu Khusheiba, providing valuable data that can inform future exploration strategies and deepen our understanding of mineral deposition in similar geological settings. This research not only contributes to the scientific community’s knowledge of the area’s geochemistry but also has potential implications for the mining and exploration industries.

## Introduction

Jordan is located along the northern boundary of the Arabian Plate, where the Arabian Nubian Shield (ANS) covers the southern part of Jordan, representing 10% of Jordan’s total area [3]. The Aheimir volcanic suite, which is a part of the Araba complex, composed of effusive and porphyritic rhyolite, and andesite rocks contains gold occurrences, within the Aheimir suite. Extensive geochemical exploration in the Wadi Abu Khusheiba area indicated that gold mineralization is present in veins up to 1 m wide and continues for several kilometres. The gold content in this vein reaches 15 g/t (grams/tonnes), whereas the gold content in stream sediment deposits of the Wadi Abu Khusheiba area reaches 40 g/t [27, 31]. The hosted rocks of the Au-bearing vein is mostly made up of xenolithic agglomerates of the Musaymir magmatic rock unit of the Aheimir suite, and these xenoliths consist of rhyolites, granites, granodiorite and tuff [3, 24,27, 31].

**Fig. 1:**
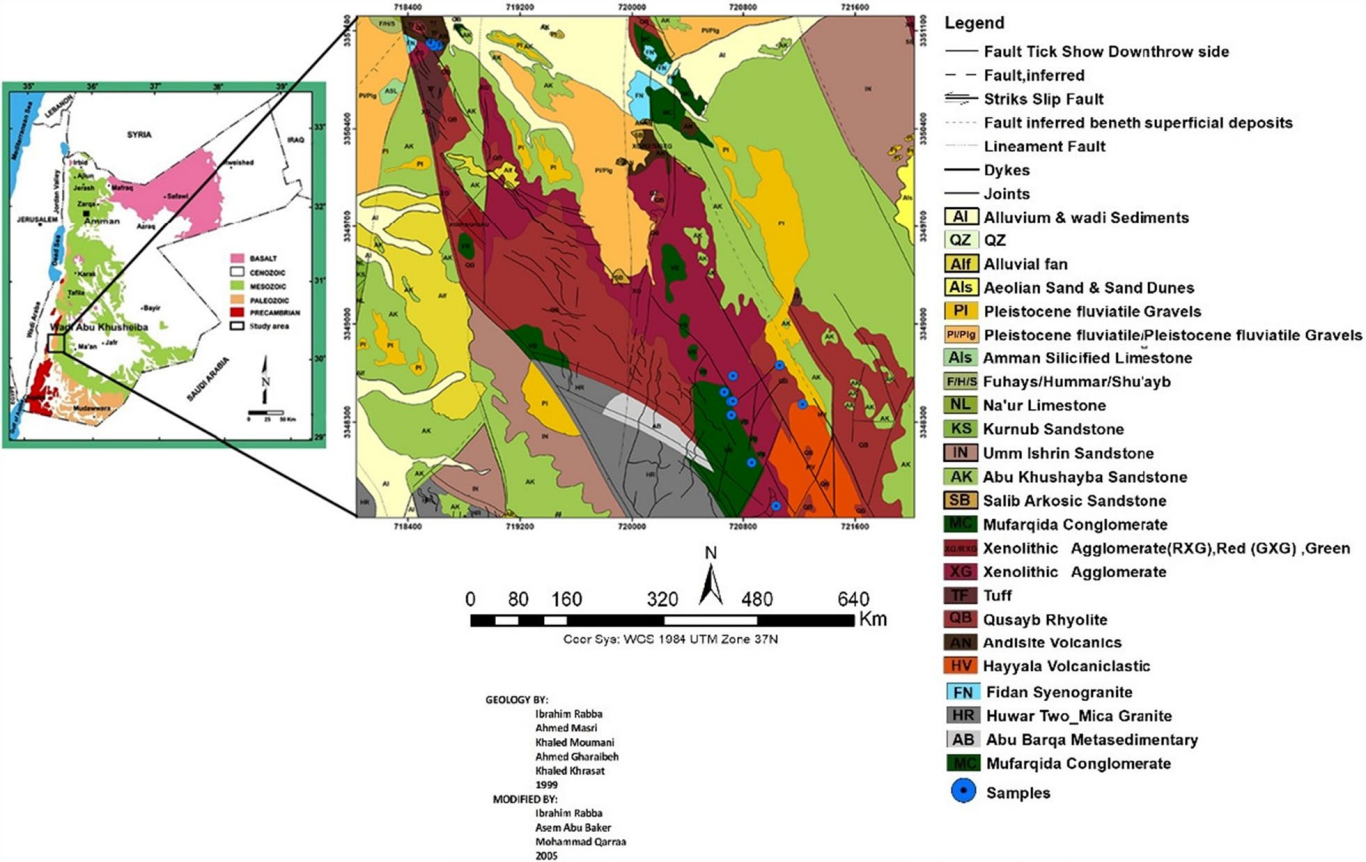
Geological map of Wadi Abu Khusheiba showing the locations of the collected samples (after [27], Fig. 2, p. 8)

The study area (Wadi Abu Khusheiba) is located in the southern part of Wadi Araba, approximately 100 km north of Aqaba and 250 km south of Amman (Fig. 1). It falls with the coordinates (UTM) of 718,000–723,000 E and 3,347,000–3,352,000 N. It is known that the gold mineralization in the study area is closely associated with hydrothermal quartz veins. Hence, a detailed geochemical study and quartz vein fracture systems can provide a better understanding of the hydrothermal processes, the geochemistry of the gold and associated elements accompanying gold mineralization [21]. Primary gold mineralization left a chemical record in the overburden and adjacent soil, most likely due to the effect of weathering processes,as a result, stream sediments might show major or minor mineralization at the local scale or across a large region [26]. Geochemical analysis of the host rock, quartz veins and stream sediments from the study area can demonstrate major and trace element anomaly patterns associated with this mineralization.

The manuscript aims to investigate the distribution and composition of gold within quartz veins in the specified region. The purpose is to understand the complex patterns of mineralization and their geological significance. This involves a detailed geochemical analysis of rock samples from the area, focusing on identifying the elemental indicators for gold and gaining insights into the geochemical behaviour of gold in this setting.

## The geology of Wadi Abu Khusheiba

The basement rocks of southwest Jordan are the northern extension of the ANS, which is split by the Red Sea Rift zone; it is composed of plutonic rocks with subsequent felsic, intermediate and basic volcanic rocks [16–19].

The basement rocks are classified as Aqaba (older) and Araba (younger) are composed of late Proterozoic igneous and metamorphic suites [14, 16, 29]. The Saramuj Conglomerate Formation represents a regional unconformity (peneplanation that separates these complexes,the Araba complex includes the Safi and Arab Mafic, as well as the Finan granitic suites and Aheimir volcanic suites [29] (Table 1). The Aheimir Volcanic Suite, which has been recognized as the principal exploration target for precious metals in the Pan African Jordanian basement, dominates the Araba Complex. The suite is oriented NNE‒SSW, occurs in a 70-km-long belt with a width of 2 to 4 kms, and is composed mainly of alkaline, lava flow, porphyritic rhyolite, with little andesite [7]. The agglomerates and rhyolites from the Aheimir volcanic suite (late Proterozoic to early Cambrian host the Au-bearing vein, which consists of four units: (1) Qusayb rhyolite, which consists primarily of large porphyritic to nonporphyritic rhyolite to microgranite., (2) Musaymir Effusive unit, which is composed of rhyolitic tuff, ignimbrites, agglomerates, volcanic breccia, and rhyolitic lava; (3) Mufarqad Conglomerate, which is primarily made up of conglomerates, with contents ranging from older Aheimir units to granites and metamorphic rocks; and (4) AlBayda Quartz Porphyry, which consists mainly of quartz, feldspar, and rhyolite porphyry rocks [4].

**Table 1:** Stratigraphic intervals showing units and suites of the Araba and Aqaba complexes and the relationship between them (based on [29]

Units	Suites	Ages/Ma
Araba complex		
AlBayda Quartz Porphyry	Aheimir Volcanic	(605–550)
Mufarqad Conglomerate		
Musaymir Effusive (host the Au-bearing vein)		
Qusayb Rhyolite		
Es Sail Aplogranite	Feinan– Humrat– Mubarak Granitic	
Fidan Syenogranite		
Humrat Syenogranite		
Mubarak Monzogranite		
Sammaniya Microgranodiorite	Araba Mafic	
Ghuweir Volcanics		
Mureihil (Umm Rachel) Diorite		
Qunaia Monzogabbro		
Haiyala Volcaniclastic	Safi group	
Saramuj Conglomerate		
Araba Unconformity (ca. 605 Ma)		
Aqaba complex		
Abu Jedda Monzogranite	Yutum Granitic	(620–605)
Imran Monzogranite		
Qara Granite		
Ishaar Granodiorite	Rumman Grano- diorite Urf Por- phyritic Suite	
Sabil Granodiorite		
Hubayra Diorite		
Mulghan Granodiorite		
Huneik Monzogranite		
Abyad Granodiorite		
Filk Monzogranite		
Rubeiq Granodiorite		
Muheirid Granodiorite		
Marsad Monzogranite		
Barraq Granodiorite		
Waara Granodiorite	Darba Tonalitic	
Muhtadi Granodiorite		
Huwwar Two Mica Granite		
Taba Monzogranite Synplutonic dikes	Rahma Foliated	
Es Sadra Granodiorite		
Umm Saiyala Granite		
Turban Granite/Granodiorite		
Abu Radmar Granodiorite		
Naba Monzogranite		
Qattar Hornblende Gabbro	Duheila Horn- blendic	

In the Aheimir volcanic suite, the stratigraphic succession reveals a complex history of volcanic activity and mineralization. The sequence begins with the Qusayb Rhyolite unit, characterized by its large porphyritic brown to dark brown rhyolite. This unit, found predominantly in the southern half of the mineralized area, represents an older phase of volcanic activity.

Overlying the Qusayb Rhyolite unit is the Musaymir Effusive unit, which is younger and holds a significant geological and economic interest due to its concentration of Au-bearing veins, particularly in the northern section of the Wadi Abu Khusheiba area. The Musaymir Effusive unit is capped by volcanic breccia, marking the youngest eruption phase within this unit. This breccia layer, extending to the top 20 m of the Musaymir Effusive unit, signifies a later stage of volcanic activity and potentially plays a role in the area’s mineralization processes.

This stratigraphic arrangement, with the older Qusayb Rhyolite unit succeeded by the younger Musaymir Effusive unit topped by volcanic breccia, outlines the volcanic evolution within the Aheimir suite. This succession is crucial for understanding the geological history and the spatial distribution of mineral resources in the region [27, 30].

## Methodology and analytical techniques

### Fieldwork and sampling

The walkout approach was used over three days to explore the research area. The very rugged topography, imposing cliffs, deep wadies, and steep slopes prevented the use of a systematic sampling strategy. A total of 28 representative samples from the mineralized vein, host rock and sediments were randomly collected from the study area, aiming to cover the whole vein as far as possible (Fig. 1). The exact locations of the samples, brief field descriptions, sample types and their related sample reference numbers are provided in (Table 2). A handheld GPS unit with a few metre accuracy was used to mark all the sample locations in the field area. Moreover, the samples contain three chip samples of the rhyolite host rock, which are labelled R1, R2, R3, R4 and R5.

**Table 2:** Location and description of the studied samples

Sample ID	Sample type	Easting	Northing	Description
R1	Host rock	718,620	3,350,996	Highly fractured weathered rhyolite with deep brown colour
R2	Host rock	718,585	3,350,994	Fractured less weathered rhyolite with salmon colour
R3	Host rock	718,562	3,351,015	Highly weathered rhyolite characterized by present of malachite (copper ore)
R4 R5	Weathered host rock	721,038 721,038	3,347,705 3,347,705	Malachite staining silicified zone, quartz fragments and silica veinlets in weathered and oxidized rhyolite
Vn1	Hanging wall	720,715	3,348,448	Hard quartz-rich felsite, compact rock with a brecciated appearance that includes silica veinlets cemented by a black substance. Colloform/crustiform textures; locally facies is pink with a cryptocrystalline patina, vein thickness is 75–85 cm
VF1	foot wall	720,715	3,348,448	
Vt1	Alteration zone	720,715	3,348,448	
Vm1	Mineralized vein	720,715	3,348,448	
Vt2	Alteration zone	720,729	3,348,450	Quartz-rich felsite Compact rock, hard, with cryptocrystalline to microcrystalline, with brecciated matter and pink colour. Colloform/crustiform textures
Vn2	Hanging wall	720,729	3,348,450	
Vm2	Mineralized vein	720,729	3,348,450	
VB3	Brecciated vein	720,729	3,348,463	Rock compact, quartz-rich felsite microcrystalline, with salmon colour Colloform/crustiform textures, with brecciated matter Thickness of vein (1-3m)
Vh3	Host rock	720,729	3,348,463	
Vm3	Mineralized vein	720,717	3,348,351	
Vm4	Mineralized vein	720,717	3,348,351	Quartz-rich felsite Compact rock, hard, with cryptocrystalline to microcrystalline, with brecciated matter and pink colour and in this station the vein become more oxidized. Colloform/crustiform textures
Vh4	Host rock	720,717	3,348,351	
Vf4	foot wall	720,717	3,348,351	
Vm5	Mineralized vein	720,835	3,348,049	Quartz-rich felsite Compact rock. Colloform/crustiform textures locally brecciated milky and dark grey coloured silica veinlets rich vein, 60 cm thickness
Vh5	Host rock	720,835	3,348,049	
Vt3	Alteration zone	720,729	3,348,463	
Vm6	Mineralized vein	720,863	3,348,014	Quartz rich Brecciated hard compact rock, dark grey colour thickness of vein approximately 40 cm
Vh6	Host rock	720,863	3,348,014	
Vf6	foot wall	720,863	3,348,014	
S1	Stream sediment	721,063	3,348,707	All the stream-sediment samples were sieved by mesh less than 2 mm (mesh 10) during the sampling in the field. The sampling was done at 30 cm depth to avoiding the organic material, contamination and any foreign material
S2	Stream sediment	721,227	3,348,428	
S3	Stream sediment	720,668	3,348,515	
S4	Stream sediment	720,510	3,348,750	

### Field observations

**Fig. 2:**
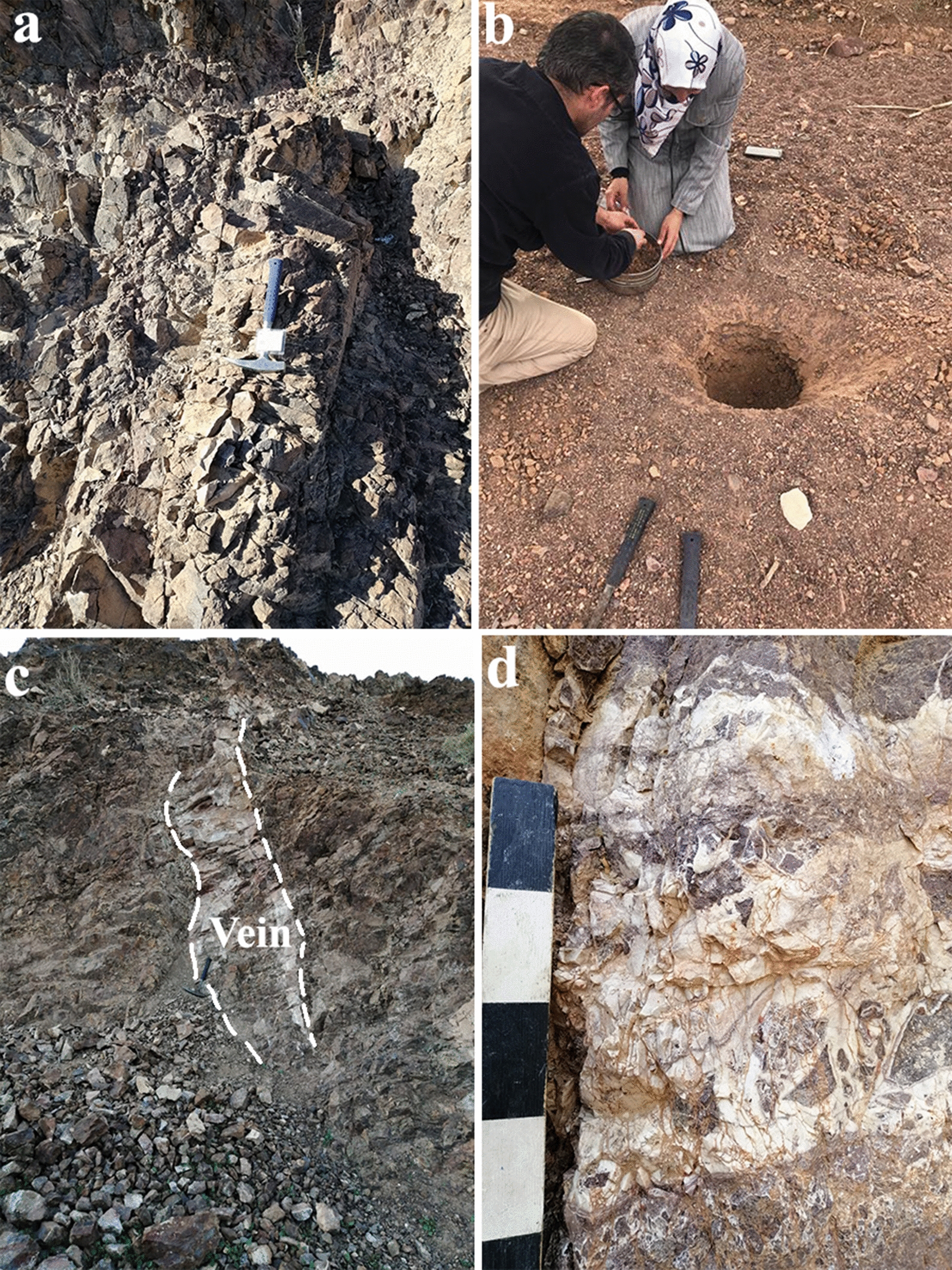
**a** The rhyolite host rocks. **b** Sampling of stream sediments. **c** The main quartz vein. **d** The brecciated nature of the vein rocks, photos by Mosleh and Al-Saudi)

Showed that the host rock, in some places, was composed of fractured porphyritic dark brown rhyolite, while in other places, the rhyolite was characterized by a pink colour (Fig. 2a). In addition, some rhyolite rocks contain copper ore, which can be distinguished by the presence of a green colour in the samples. Within the research area, four stream sediment samples were collected from the B horizon; after digging up to a depth of 30 cm, fresh samples were bagged to avoid the introduction of organic material, contamination and unwanted materials. This was achieved through digging using a stainless steel hand auger, and then the collected sample was placed directly into cloth bags. Approximately 5 kg of sediment was collected from each location. All stream sediment samples were sieved using a mesh less than 2 mm (mesh 10) in size during sampling in the field, and the samples were then labelled S1, S2, S3 and S4 (Fig. 2b).

Finally, a total of 19 samples were obtained along the significant quartz vein. The vein tracked sporadically for a total of approximately 800 m, and it has a clearly visible 0.3–1 m horizontal thickness (Fig. 2c). The main host rock is quartz-rich rhyolite compact, which is a hard rock with a brecciated nature (Fig. 2d). Several rock samples of quartz vein were cut and polished for petrography study. Thin section slides were prepared following the procedure outlined in Hutchison [12]. Subsequently, these slides were studied, examined, and photographed using a Leica polarizing and reflected photographic microscope equipped with a digital camera. This work was carried out at the Laboratories of the Faculty of Archaeology and Anthropology, Yarmouk University, Jordan.

### FESEM-EDX

A field emission scanning electron microscopy (JEOL JSM-7600F)—energy dispersive X-ray (OXFORD X-Max) (FESEM-EDX) technique and specific surface area analysis were applied on polished sections of 9 representative different samples for semiquantitative chemical analysis.

The authors have performed this examination to provide an extra confidence regarding the outcomes related to trace elements. The samples were investigated at the Faculty of Science at the Universiti Malaya, Malaysia, for detailed geochemical analysis to determine the chemical composition, identify the ore and gangue minerals and reveal information about their textures.

### Inductively coupled plasma‒mass spectrometry (ICP‒MS)

Inductively coupled plasma‒mass spectrometry (ICP‒MS) was used to determine trace element content in the 28 samples; powdered samples were analysed at the Jordan Atomic Energy Commission using inductively coupled plasma‒mass spectrometry (Agilent 7500a), According to Winefordner [37] the samples were prepared as follows:

In a polytetrafluoroethylene (PTFE) crucible, 0.1 g of sample was wetted with 3 mL of distilled water. In a fume hood, 4 mL of an acid combination (HF: HClO4 = 5:1) was added to the crucible and then heated to 260 °C on an electric hot plate digestion system. Another 4 mL of the acid mixture was heated until no more white smoke was emitted. After that, 2 mL of aqua regia (HCl: HNO3 = 3:1) was added to redissolve the mixture. The sample solution became clear and transparent after the addition of 10 mL of 10% aqua regia to the extract. After the solution was cooled, 2 mL HNO_3_ was added, and finally, the sample extract was transferred to a 100 mL PTFE volumetric flask with distilled water to adjust the volume. Analysis was conducted using the Accu Standard, which is pure for gold (Au) and silver (Ag), as well as a multielement standard solution for other elements.

### Atomic absorption spectroscopy (AAS)

An atomic absorption spectroscopy (AAS) technique was utilized to analyse the major elements at the Laboratories of the Faculty of Archaeology and Anthropology at Yarmouk University, Jordan. Analytikjena—ContrAA 800 type was used, and calibration was performed via the use of a Certipur ICP multielement standard solution IV (111,355). The powders of 24 rock samples were transformed to solutions according to a method by Hesse [13] to determine the major elemental content. These data on the major elements are essential for classifying and identifying the rocks.

### Accuracy and precision of AAS analysis

In the geochemical analysis of our samples using Atomic Absorption Spectroscopy (AAS) at the Laboratories of the Faculty of Archaeology and Anthropology at Yarmouk University, we employed rigorous calibration and quality control measures. The AAS instrument (Analytikjena—ContrAA 800 type) was calibrated using the Certipur ICP multielement standard solution IV (111,355) prior to analysis. This calibration was repeated at regular intervals to ensure consistent accuracy throughout the analytical process. The detection limits for each element analyzed were established based on the instrument's specifications and the concentration levels expected in our samples. These limits are crucial for accurately interpreting trace element concentrations in the study. To ensure the precision of our analysis, replicate analyses were conducted on selected samples. The results of these replicates were used to calculate the standard deviation, providing a quantitative measure of the method’s precision. Quality assurance was further reinforced through the use of blanks and certified reference materials (CRMs). The inclusion of these quality control samples in each batch of analyses served to monitor any potential contamination and to verify the accuracy of our results. We also performed statistical analyses of the data to assess the precision and accuracy comprehensively. These analyses included calculating the relative percent difference between duplicates and comparing our results to established literature values where available. Potential sources of error, such as instrument drift, matrix effects, and sample contamination, were carefully considered and addressed. Regular instrument maintenance and adherence to strict sample handling protocols were implemented to mitigate these issues.

Through these measures, we ensured that the AAS method used in our study provided reliable and accurate results, contributing to the robustness of our findings and their significance in understanding the geochemistry of the studied area.

### Statistical analysis

To perform statistical analysis of the data and create the geochemical anomaly maps, Excel 2010, Arc Map 10.8 and Arc Catalogue 10.8 programs were used, 21 samples were analysed using confidence level of 85%.

## Results

### Geochemical analyses

#### Major oxides

The composition of major oxides in the analyzed rock samples of mineralized veins and host rocks are shown in (Table 3), and the range values are as follows: SiO_2_ (77.3–82.2) wt.%, Al2O3 (7.1–13.3) wt.%, K_2_O (5.4–8.8) wt.%, Fe_2_O_3_ (0.5–3.5) wt.%, MnO (0.01–0.84) wt.%, MgO is (0.07–1.2)wt.%, Na_2_O (0.1–1.1) wt.%, CaO (0.12–1.2) wt.%, TiO_2_ (0.01–0.73)wt.%, P_2_O_5_ ( 0.03–0.06) and Cr_2_O_3_ (0.02–0.21)wt.%. This reveals that the host rock samples are quartz-rich felsite dykes.

**Table 3:** Chemical analysis of the major oxides (in wt. %) in the rock and vein samples

Sample Id	Sample type	SiO_2_%	Al_2_O_3_%	Fe_2_O_3_%	MnO%	MgO%	CaO%	K_2_O%	Na_2_O%	TiO_2%_	Cr_2_O_3_%	P_2_O_5_%	LOI	Total
R1	Host rock	77.3	13.25	1.64	0.84	0.1	0.13	5.4	1.1	0.35	0.1	0.03	0.65	99.3
R2	Host rock	78.2	11.2	3.11	0.25	0.2	0.12	6.9	0.9	0.51	0.07	0.04	0.78	99.2
R3	Host rock	80.1	10.7	2.29	0.17	0.53	0.25	6.7	0.7	0.04	0.06	0.03	0.65	99.9
R4	Weathered host rock	77.3	9.2	3.5	0.8	1.2	0.6	7.3	0.59	0.13	0.03	0.04	0.77	98
R5	Weathered host rock	78.6	9.3	0.8	0.07	0.1	0.7	6.8	0.63	0.01	0.02	0.06	2.2	98.5
Vn1	Hanging wall	81.1	8.7	0.5	0.02	0.2	0.4	6.4	0.22	0.01	0.17	0.05	0.88	98.2
VF1	foot wall	82.2	8.1	1.1	0.09	0.1	1	6.3	0.4	0.01	0.19	0.05	0.73	99.2
Vt1	Alteration zone	80.2	9.1	2.4	0.07	0.3	1.1	7.1	0.7	0.73	0.09	0.05	0.34	99.8
Vm1	Mineralized vein	82	8.2	0.9	0.03	0.2	0.4	6.4	0.2	0.21	0.21	0.06	0.47	98.4
Vt2	Alteration zone	79.7	8.9	3	0.03	0.4	0.7	6.7	0.19	0.18	0.15	0.03	2.6	99.6
Vm2	Hanging wall	79	9	2.2	0.04	0.1	0.3	9	0.11	0.22	0.17	0.06	1.7	99.7
Vn2	Mineralized vein	78.6	10	1.5	0.04	0.1	0.7	6.8	0.35	0.43	0.11	0.04	0.86	98
VB3	Brecciated vein	77.5	11	0.7	0.02	0.1	0.3	8.8	0.21	0.54	0.19	0.05	0.77	99.5
Vh3	Host rock	81	8.1	2.5	0.1	0.5	0.3	6.6	0.61	0.18	0.17	0.03	1.84	99.4
Vm3	Mineralized vein	79.1	9.4	1.2	0.3	0.7	0.4	7.4	0.21	0.15	0.17	0.03	0.94	98.8
Vm4	Mineralized vein	80.3	9.3	1.6	0.01	0.07	0.4	7.8	0.61	0.19	0.25	0.05	0.87	99.9
Vh4	Host rock	79.3	10.8	0.9	0.07	0.1	1	6.1	0.12	0.34	0.17	0.03	1.87	99.9
Vf4	foot wall	80.1	9.6	1.1	0.04	0.2	0.9	6.7	0.17	0.12	0.09	0.04	0.77	98.7
Vm5	Mineralized vein	77.9	11.1	2.6	0.03	0.8	0.7	7.2	0.15	0.63	0.21	0.03	0.89	99.6
Vh5	Host rock	78.7	9.2	1.6	0.8	1	0.7	5.8	0.35	0.14	0.18	0.05	1.8	98.7
Vt3	Alteration zone	78.4	9.4	3.1	0.5	0.7	1	6.7	0.23	0.3	0.07	0.04	1.3	98.6
Vm6	Mineralized vein	77.8	11.9	3.5	0.1	1	1.1	5.7	0.5	0.18	0.18	0.05	0.97	99.5
Vh6	Host rock	78.7	9.2	3.1	0.1	1.2	1.2	6.5	0.1	0.56	0.06	0.05	1.3	99
Vf6	foot wall	77.8	11.1	2.6	0.1	0.8	1.2	6.7	0.25	0.22	0.02	0.03	0.78	99

#### Trace element results

The concentrations of the trace in ppm in the analysed samples are documented in (Table 4). In our geochemical analysis, special attention was given to maintaining the integrity and relevance of the data by distinctly separating mineralized samples from other types of samples, including host rocks and stream sediments. This separation is critical in geochemical studies to avoid mixing data from different sources, which could otherwise lead to inaccurate interpretations.

**Table 4:** Chemical analysis of trace elements (in ppm) in the rock, vein and stream sediment samples.

Sample Id	Sample types	Au(ppm)	Ag(ppm)	Cu(ppm)	Pb(ppm)	As(ppm)	Ba(ppm)	Sb(ppm)	Mo(ppm)	Zn(ppm)
R1	Host rock	< 1	< 1	66	11	5	220	0.6	2	54
R2	Host rock	< 1	< 1	51	4	6	60	0.8	1.8	66
R3	Host rock	< 1	< 1	**30,240**	**151**	**8**	**6996**	0.41	1.6	39
R4	Weathered host rock	< 1	0.06	5228	13	**9**	1259	0.9	1.35	66
R5	Weathered host rock	0.001	0.96	2030	15	**11.2**	1310	1.14	1.32	67
Vn1	Hanging wall rock	**5**	**18**	293	12.5	4.1	480	1.23	4.5	63
VF1	foot wall	1.8	5.6	80	4	5	370	1.1	**5.1**	70
Vm1	Mineralized vein	**3**	**9.2**	525	9	3	400	**2.2**	4.3	50
Vt1	Alteration zone	< 1	< 1	811	7	4.5	440	1.2	**4.8**	65
Vt2	Alteration zone	< 1	0.02	25	45	2.7	620	1.01	2.1	**990**
Vm2	Mineralized vein	1.2	2.3	30	56.6	3.6	800	1.02	2.1	**1590**
Vn2	Hanging wall	0.8	1.7	32	41	4.2	820	1.02	1.9	**1340**
VB3	Brecciated vein	0.005	6.02	45	25	3.2	420	0.23	0.22	41
Vh3	Host rock	< 1	0.001	48	18	2.7	330	0.27	0.35	35
Vm3	Mineralized vein	0.009	0.03	43	22	3.3	470	0.28	0.38	40
Vm4	Mineralized vein	0.717	1.61	30	**147**	1.5	590	0.88	2.21	32
Vh4	Host rock	0.320	0.02	25	80	1.3	630	0.78	2.13	28
Vf4	foot wall	0.11	0.001	30	77	1.36	490	0.76	1.97	35
Vm5	Mineralized vein	**4.27**	2.27	24	8.6	5	1370	**2.04**	3.5	39
Vh5	Host rock	1.2	2.34	28	7.7	4	1260	**2.01**	2.22	41
Vt3	Alteration zone	0.007	1.3	23	8.9	2.6	2214	**2.06**	1.98	33
Vm6	Mineralized vein	0.004	0.22	13.1	10	2.6	1140	0.68	2.22	78
Vh6	Host rock	< 1	0.005	11.5	7	1.9	1132	0.55	2.14	80
Vf6	foot wall	< 1	0.007	10.6	8.9	2.1	1201	0.65	2.22	79
S1	Stream sediment	0.0009	0.017	20	15	3.23	425	0.172	0.34	17
S2	Stream sediment	0.0012	0.25	22	15	2.87	337	0.183	0.58	13.8
S3	Stream sediment	0.0025	0.027	14.3	7.32	1.51	473	0.115	0.81	20.6
S4	Stream sediment	0.0011	0.014	9.24	6.52	1.59	591	0.151	0.35	13

Bold markings indicate statistically anomalous values for specific elements in each sample

Upon isolating the mineralized samples, we calculated the average concentrations of various trace elements. The results are as follows:

- Mineralized vein samples: Gold (Au): 0.83 (ppm), Silver (Ag): 1.7ppm, Copper (Cu): 142.6 ppm. Lead (Pb): 37.2ppm, Arsenic (As): 3.47ppm, Barium (Ba): 750 ppm, Antimony (Sb):0.9 ppm, Molybdenum (Mo): 2.17 ppm, Zinc (Zn):42.1 ppm.
- Host rock samples: Gold (Au): 0.17 parts per million (ppm), Silver (Ag): 0.38ppm, Copper (Cu): 4191.9 ppm, Lead (Pb): 34 ppm, Arsenic (As): 5.5 ppm, Barium (Ba): 1466.3 ppm, Antimony (Sb): 0.61ppm, Molybdenum (Mo): 5.05 ppm, Zinc (Zn): 52.9 ppm.
- Footwall samples: Gold (Au): 0.64 (ppm), Silver (Ag): 1.87 ppm, Copper (Cu): 40.2 ppm, Lead (Pb): 30 ppm, Arsenic (As): 2.82ppm, Barium (Ba): 2.82ppm, Antimony (Sb): 0.84 ppm, Molybdenum (Mo): 3.1 ppm, Zinc (Zn) 61.3.
- Hanging wall samples: Gold (Au): 2.9 (ppm), Silver (Ag): 9.85ppm, Copper (Cu): 163 ppm, Lead (Pb): 26.8 ppm, Arsenic (As): 4.15ppm, Barium (Ba): 650 ppm, Antimony (Sb): 1.13 ppm, Molybdenum (Mo): 3.2ppm, Zinc (Zn) 702.
- Alteration zone samples: Gold (Au): 1.0 (ppm), Silver (Ag): 3.51ppm, Copper (Cu): 191 ppm, Lead (Pb): 21 ppm, Arsenic (As): 2.77ppm, Barium (Ba): 1078 ppm, Antimony (Sb): 1.76ppm, Molybdenum (Mo): 2.79ppm, Zinc (Zn) 358.
- Stream sediment samples: Gold (Au): 0 (ppm), Silver (Ag): 0.08ppm, Copper (Cu): 16.4 ppm, Lead (Pb): 11 ppm, Arsenic (As): 2.3ppm, Barium (Ba): 457 ppm, Antimony (Sb): 0.16 ppm, Molybdenum (Mo): 0.52ppm, Zinc (Zn) 16.1.

These average concentrations reflect the specific geochemical signature of the mineralized samples within our study area. They provide crucial insights into the elemental makeup of the mineralization, which is essential for understanding the deposit's genesis, as well as its economic potential.

The highest Au contents were found in samples Vn1 and Vm5, at 5 ppm and 4.27 ppm, respectively. The highest levels of Ag were found in the Vn1 and Vm1 samples, which were 18 ppm and 9.2 ppm, respectively. Moreover, vein samples Vm2, Vt2, Vn2, Vm4, Vh4, and Vf4 contained high amounts of Pb, Zn, Cu and Ba. Rock sample analysis revealed negligible levels of antimony (usually < 2.5 ppm), molybdenum, and silver (< 2 ppm). Only samples Vn1, VF1, Vm1, and Vt1 showed values of 4.5, 5.1, 4.3, and 4.8 ppm for molybdenum, and only samples Vn1, Vm1, Vf1 and Vb3 had elevated values of silver (18, 9.2, 5.6 and 6.02 ppm, respectively). The maximum amount of arsenic observed was a few tens of ppm.

#### Field emission scanning electron microscopy

**Fig. 3:**
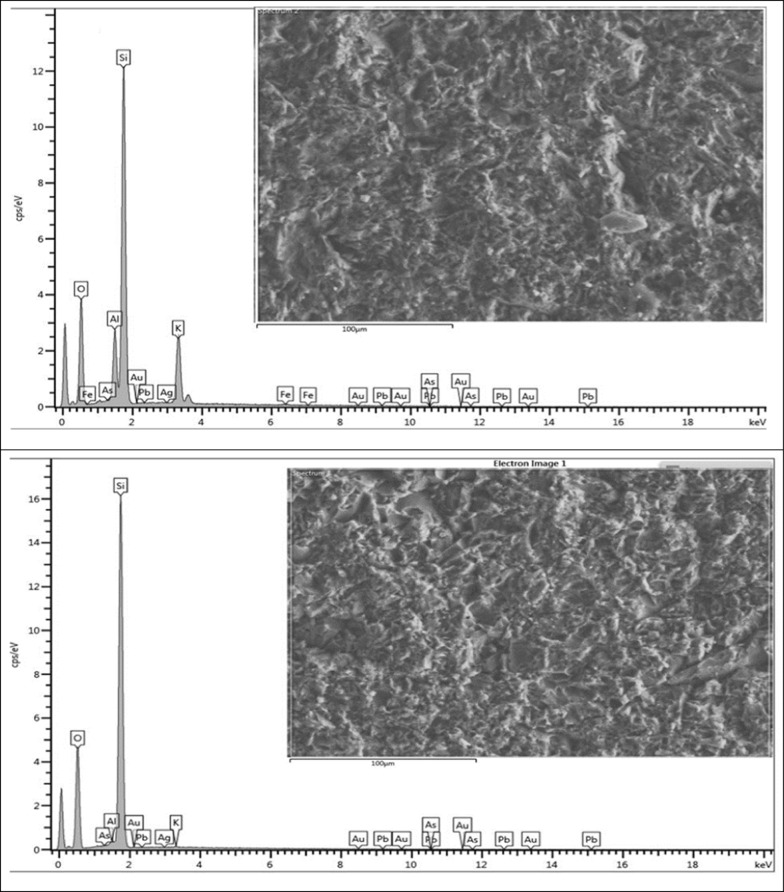

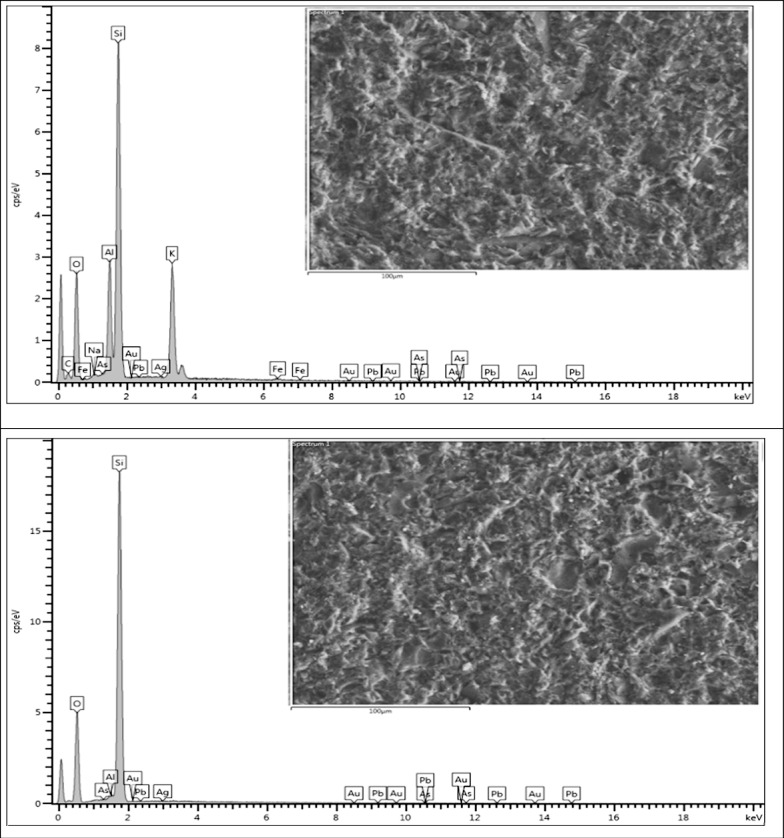
Qualitative results of specific surface area analysis of polished section for different selected samples using (FESEM-EDX)

The results of mineral chemical analysis of polished sections for selected representative samples of vein rocks for which area analysis was applied using the FESEM-EDX technique are given in (Table 5, Fig. 3). The results indicate that samples Vn1 and Vf1 contain traces of Au (0.01%). Ag is found in samples Vm4, Vm5, and Vf1 at concentrations of 0.06%, 0.03%, and 0.02%, respectively. Zn is present in sample Vn5 at 0.09%, and Pb is detected in sample Vm4 at 0.05%. Additionally, traces of As are observed in samples Vb3, Vm2, Vm3, Vm4, and Vm5, with concentrations of 0.16%, 0.01%, 0.03%, 0.11%, and 0.05%, respectively.

**Table 5:** FESEM-EDX semiquantitative chemical analysis for select representative samples of vein rocks; the results are provided in wt.%

Sample Id	Sample type	O	Al	Si	K	As	Mg	Ag	Au	Pb	Fe	Ca	Cu	Zn	Na
Vn1	Hanging wall	65.6	0.44	33.85	0.09	0.01	0	0	0.01	0.01	0	0	0	0	0
Vf1	Foot wall	62.69	7.06	22.72	7.44	0	0	0.02	0.01	0.04	0.08	0	0	0	0
Vm2	Mineralized vein	65.55	0.59	33.86	0	0.01	0	0	0	0	0	0	0	0	0
Vb3	Brecciated	53.03	5.73	32.46	2.86	0.16	0	0	0	0	1.29	0.99	0.11	0	2.94
Vm3	Mineralized vein	50.85	8.2	28.89	10.97	0.03	0	0	0	0	0.61	0.02	0	0	0.36
Vm4	Mineralized vein	53.23	4.76	34.42	5.65	0.11	0	0.06	0	0.05	1.05	0.18	0	0	0.48
Vm5	Mineralized vein	61.67	7.67	22.15	7.94	0.05	0	0.03	0	0	0	0	0	0	0.37
Vn5	Hanging wall	52.88	7.21	27.98	5.93	0	0.26	0	0	0	1.5	0.24	0	0.09	1.85
Vh5	Host rock	54.67	7.92	27.85	4.67	0	0.31	0	0	0	0.72	0.39	0.39	0	3.03

#### Petrography analysis

**Fig. 4:**
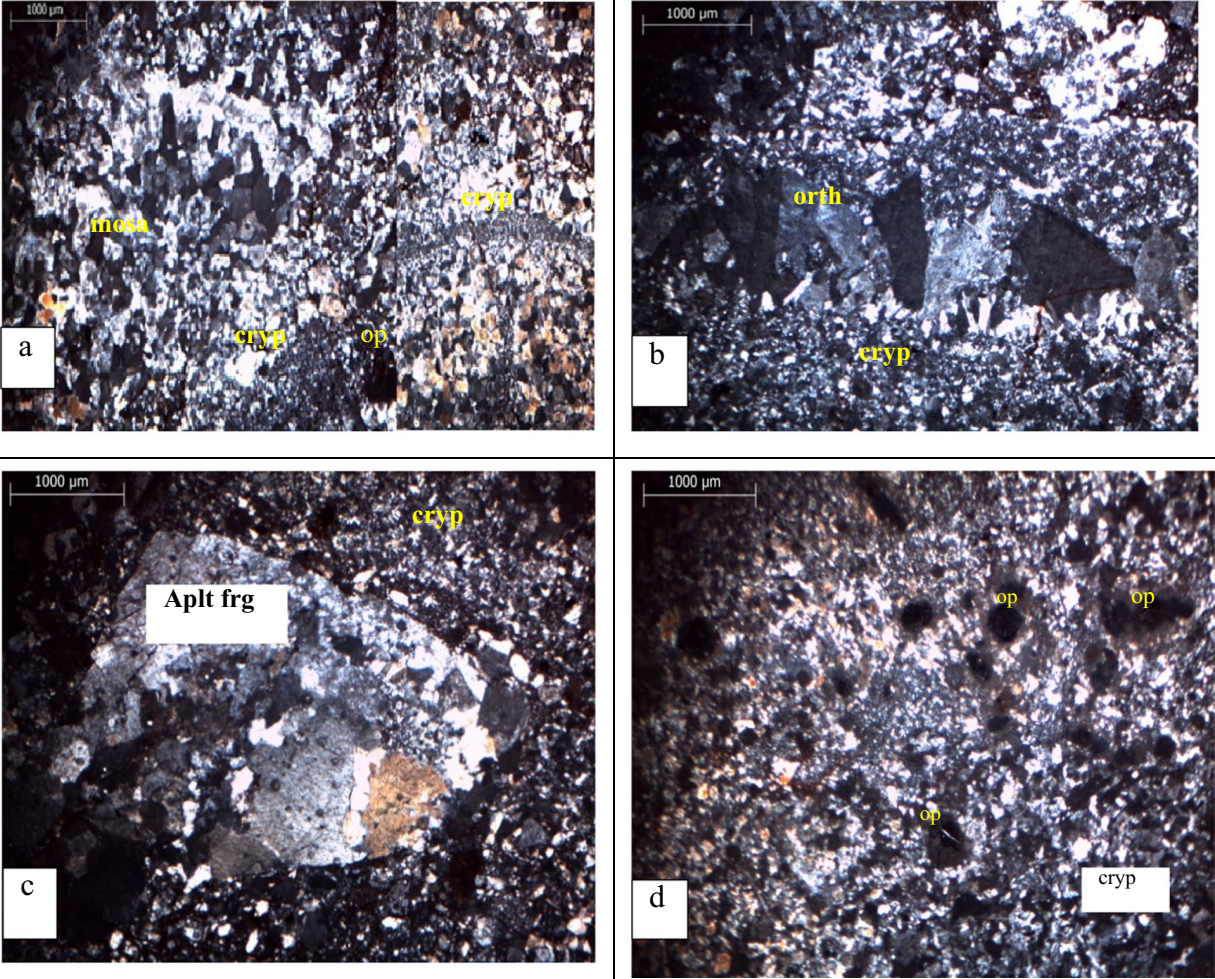
polarized light photomicrographs (XPL) showing the primary minerals of the rock chip samples. **a** mosaic and cryptocrystalline Qz. **b** orthoclase. **c** Aplite fragments with cryptocrystalline Qz, **d** Opaque minerals. Aplt frg = aplite fragment, op, Opaque; orth, orthoclase; cryp, cryptocrystalline; Qz, Quartz

**Fig. 5:**
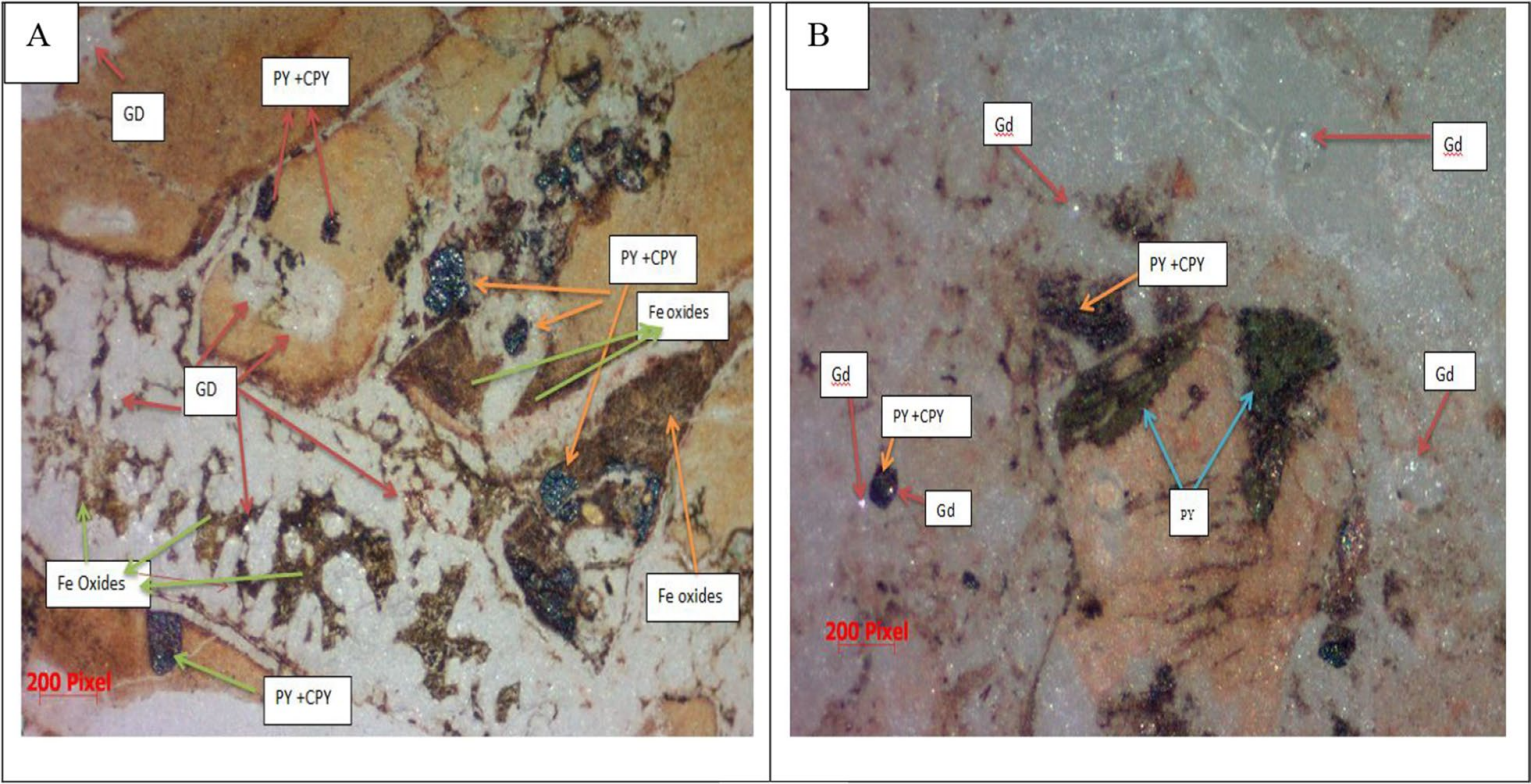
Reflected light photomicrographs showing the Ore minerals composition. Gd = Gold, py = pyrite, cpy = chalcopyrite

Thin section petrographic analysis were conducted to determine primary minerals as well as secondary minerals, The petrographic analysis of the vein rock chip samples yielded the following results: In the investigated rock samples, quartz is most abundant as primary and as a replacement mineral, occurring in every sample studied, it is found as a mosaic of irregular, fine-grained, microcrystalline aggregates and cryptocrystalline grains, with anhedral to subhedral crystal forms (Fig. 4a). Alkali feldspar, which primarily consists of orthoclase, is the second primary mineral in all vein rocks that have been studied (Fig. 4b). The vein rock samples sections materializes a brecciated facies which consists of fragments of rocks, the fragments of rock comprise aplite fragments (abundant) (Fig. 4c), the various fragments are cemented by a cryptocrystalline material (black material), probably devitrified volcanic glass of quartzo feldspathic composition, strongly impregnated by fine opaque minerals and characterized by present of dark brown veins and cracks that filled with iron oxides opaque minerals that observed in polarized microscope considered as accessory minerals in the studied vein rock samples (Fig. 4d). Mineragraphy analysis results by Leica reflected microscope show that the ore minerals seen in the polished section are mainly pyrite, chalcopyrite, gold, iron oxides (Fe- oxides), the appearance of these minerals is shown in (Fig. 5). Gold appears as very fine disseminated granular grains, it is displayed in a quartzose gangue at the free State, and the samples under study show micron-scale dimensions of it, this native gold has a yellow colour on a microscopic scale and particularly in natural light.

In the aplitic granite fragments, pyrite was widely dispersed. Pyrite aggregates are frequently found in vugs and open voids; goethite may have been created wholly or partially from pyrite. The micronic granules of chalcopyrite have interstitial insulation, and some of the grains exhibit a complete alteration.

## Discussion

### Major oxides and classification of volcanic rocks

**Fig. 6:**
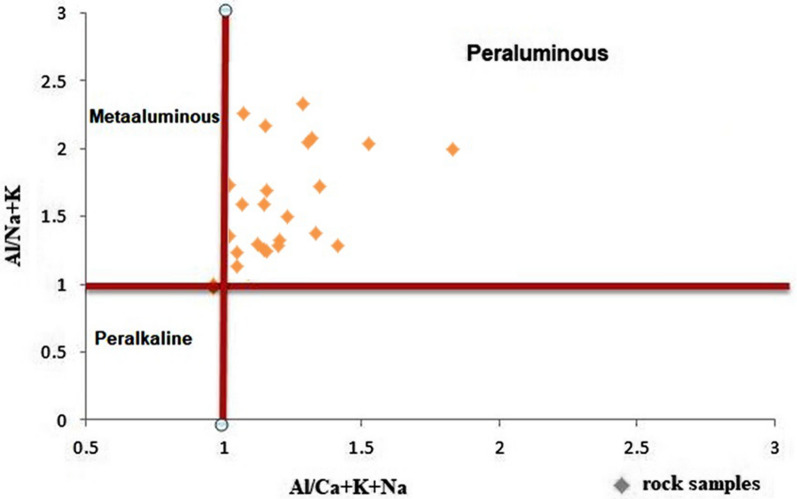
A/NK versus A/CNK Shand diagram (after [23]) )A/CNK, molar Al_2_O_3_/(CaO + Na_2_O + K_2_O); ASI, aluminium saturation index; A/NK,  molar Al_2_O_3_/(Na_2_O + K_2_O)

**Fig. 7:**
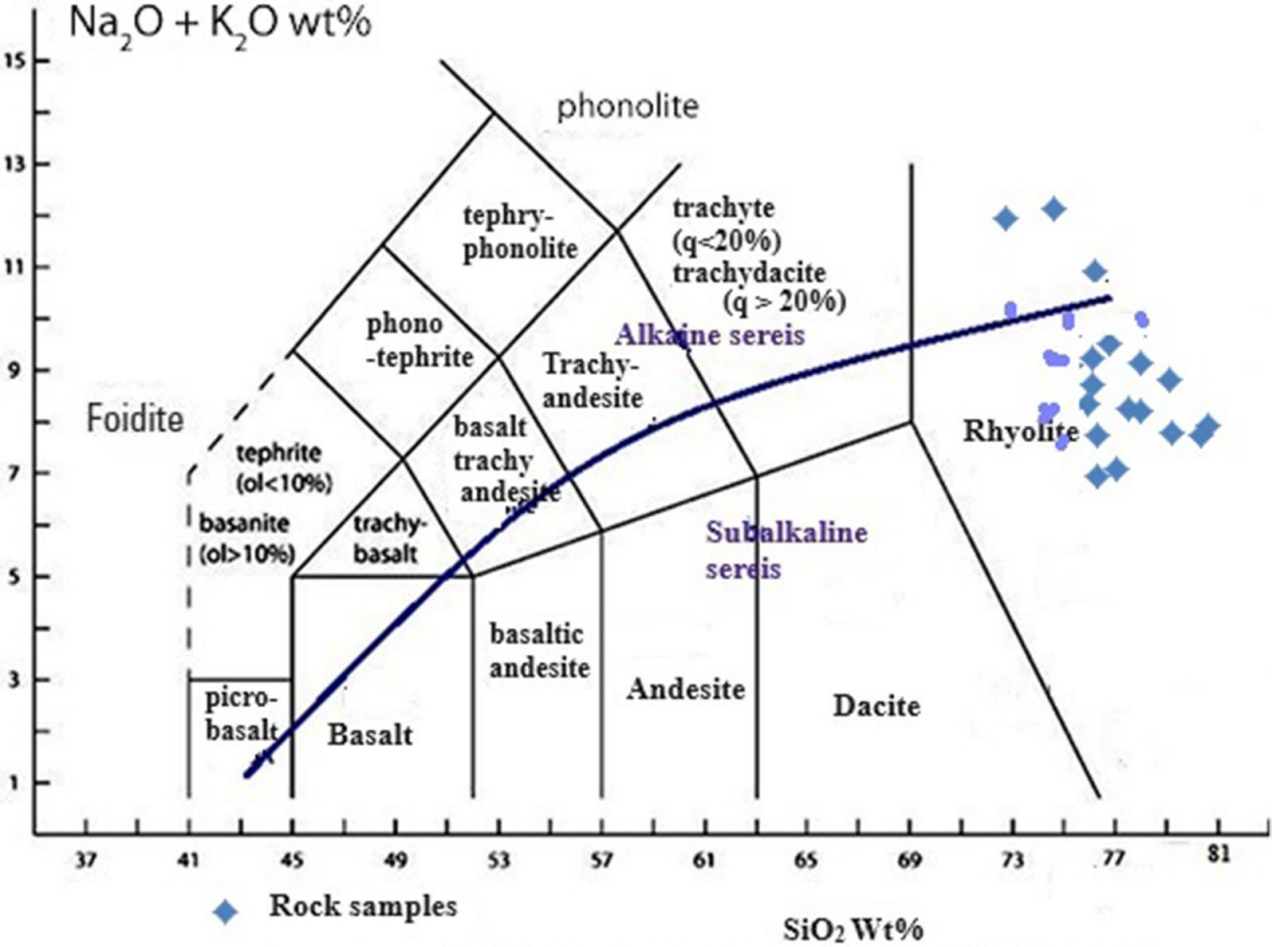
Total alkali silica (TAS) diagram in wt. %, as shown by Bas et al. [5] and formalized by Le Maitre [22], the blue curved line between the alkaline and subalkaline series is that of Irvine and Baragar [15]

Samples of the rocks under study include more than 66% SiO_2_ and may be identified as silica oversaturated rocks based on free silica according to Shand [33]. Figure 6 shows that the vein samples in this investigation display peraluminous properties, according to the alumina saturation index value proposed by Shand [32]. The samples that were examined all fall within the rhyolite field, as per the chemical categorization of total alkali silica [10, 22] (Fig. 7). This data is in agreement with what has been found in the Wadi Abu Khusheiba area by Okour et al. [27] and Al-Hwaiti et al. [2]. The former group found that the veins are located within felsic volcanic rocks, while the latter group found that the majority of their samples plot in the rhyodacite/dacite field. Al Khawalde [1] states that the Aheimir volcanic suite is divided into two main phases. The first phase consists of felsic volcanic rocks with high silica and alkalis contents,this suite includes effusive rocks like rhyolitic lava flows, extrusive rocks like pyroclastic and ignimbrite, and minor intrusive rocks like microgranites. The majority of the volcanic samples that have been studied fall into the rhyolite fields.

### Geochemical anomalies and correlation

**Fig. 8:**
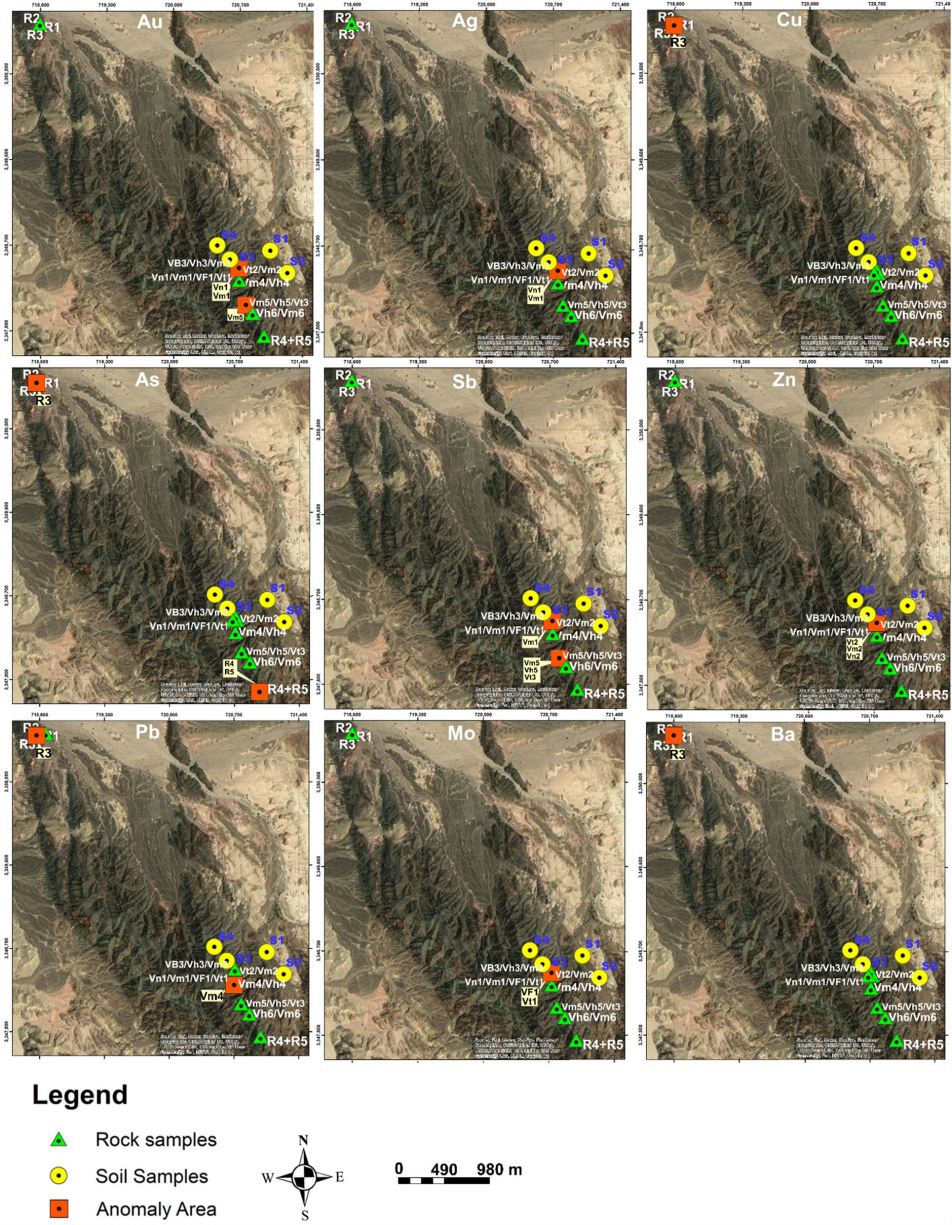
Satellite images showing the distribution of Au, Ag, Cu, As, Sb, Pb, Zn, Ba, and Mo anomalies in the study area

Anomalies in the geochemistry of Au, Ag, Cu, Pb, As, Ba, Sb, Mo, and Zn were found by means of elementary classical statistical tests (Table 6). Statistics were performed using two threshold values, with threshold 1 being the mean plus two standard deviations (SD) and threshold 2 being the median plus two SD, with the background values being used as the mean. To create geochemical anomaly maps, values that are considered abnormal are highlighted in bold (Fig. 8). The rock samples’ Pearson’s correlation coefficients for these element concentrations are shown in Table 7. Weak positive linear correlations were found between the concentrations of Au and as (r = 0.1), as well as strong positive correlation between Au and Sb (r = 0.51), Au and Ag (r = 0.75), and Au and Mo (r = 0.77), suggesting common sources. Negative correlations were observed for Au with Cu, Pb, Ba, and Zn, Ag with Cu, Pb, Ba, and Zn, Cu with Pb, Mo, and Zn, As with Zn, Ba with Mo and Zn, Sb with Zn, and Mo with Zn, suggesting distinct sources. The results are consistent with those of other researchers, such as Okour et al., [27], Al-Hwaiti et al., [2], Oke et al., [26], Bany Yaseen [38, 39], Ernawati et al., [11], and Stergiou et al., [34], who have reported similar findings in different areas.

**Table 6:** Statistical summary of select elements in the rock vein samples and stream sediment samples

	Au(ppm)	Ag(ppm)	Cu(ppm)	Pb(ppm)	As(ppm)	Ba(ppm)	Sb(ppm)	Mo(ppm)	Zn(ppm)
Min	0.0009	0.001	9.24	4	1.3	60	0.115	0.22	13
Max	5	18	30,240	151	11.2	6996	2.2	5.1	1590
Mean	0.97	2.17	1421.7	29.75	3.82	958.85	0.87	2.01	181.62
Median	0.11	0.23	30	12.75	3.21	590.5	0.79	1.99	45.5
std. Dev	1.33	3.87	5742.51	39.49	2.37	1272.49	0.606	1.36	405.74
threshold-1	3.62	9.9	12,906.72	108.74	8.56	3503.85	2.09	4.74	993.12
threshold-2	2.76	7.97	11,515.02	91.74	7.94	3135.5	2	4.71	856.1

**Table 7:** Correlation matrix of the trace elements in rock vein samples (Total samples 21 samples with 85% confidence level)

	Au	Ag	Cu	Pb	As	Ba	Sb	Mo	Zn
Au	1	0.75	− 0.05	− 0.29	0.1	− 0.02	0.51	0.77	− 0.05
Ag		1	− 0.09	− 0.2	0.05	− 0.32	0.3	0.6	− 0.1
Cu			1	− 0.18	0.74	0.21	0.01	− 0.11	− 0.13
Pb				1	− 0.04	− 0.29	− 0.23	0.19	0.2
As					1	0.25	0.18	0	− 0.02
Ba						1	0.44	− 0.15	− 0.07
Sb							1	0.56	− 0.03
Mo								1	− 0.08
Zn									1
SiO_2_%	0.37	0.42	− 0.25	0.13	− 0.2	− 0.63	0.04	0.58	− 0.1
Al_2_O_3_%	− 0.23	− 0.24	− 0.16	0.1	− 0.2	0.32	− 0.10	− 0.27	− 0.1
Fe_2_O_3_%	− 0.02	− 0.24	0.21	− 0.23	− 0.1	0.52	0.13	− 0.15	0.11
MnO%	− 0.20	− 0.2	0.52	− 0.29	0.3	0.51	0.28	− 0.23	− 0.2
MgO%	0.021	− 0.34	0.28	− 0.48	0.04	0.56	0.06	− 0.2	− 0.3
CaO%	− 0.09	0.2	− 0.11	− 0.23	− 0.2	0.39	0.24	0.26	− 0.2
K_2_O%	− 0.07	0.1	0.08	0.31	0.05	− 0.16	− 0.19	− 0.34	0.37
Na_2_O%	− 0.05	0.04	0.43	0.01	0.4	− 0.05	0.10	− 0.05	− 0.2
TiO_2%_	0.20	0.02	− 0.27	− 0.24	− 0.3	− 0.24	0.24	0.02	0.03
Cr_2_O_3_%	0.24	0.21	− 0.29	0.14	− 0.4	− 0.32	0.06	0.25	0.07
P_2_O_5_%	− 0.01	0.36	− 0.05	− 0.05	0.32	− 0.05	0.17	0.34	0.08

It turns out that the dataset’s trace element relationships are more complicated than we thought. It seems that the presence of other trace elements in your samples is independent of Au, since there is no substantial association between the two. Observable shifts in the relative abundances of the many elements do not follow from variations in the concentration of a single element. The fact that the significance threshold varies with the confidence level suggests that there may be weak associations, but they need more statistical proof to be deemed significant. Reasons for this can include things like very constant element concentrations or something else entirely. A lack of relationships that are statistically significant does not prove total independence. Understanding the environmental or geochemical processes at play requires looking at possible weak links while keeping the context in mind.

There seems to be only a very faint positive association between the concentrations of gold (Au) and arsenic (As) in your samples, as shown by the correlation coefficient of 0.1, which indicates that the relationship is strong. This indicates that having knowledge about the concentration of one element offers very little information about the concentration of the other element that is likely to be there. If you are employing a high degree of confidence, such as 95% or 99%, you will need more convincing evidence to reject the null hypothesis, which states that there is no association between the two variables. It is possible that a correlation of 0.1 does not satisfy these criteria. An assortment of elements, including geological processes, mineral associations, and sampling methodologies, have the potential to have an impact on the quantities of gold and arsenic found in natural samples. These natural variations have the potential to mask weak links.

### Trace element analyses

The trace element analysis results indicate the highest level of Pb in Vm4, at 147 ppm. Lead exists in a divalent state (Pb^+2^) with an ionic radius approximately similar to K^+^. As a result, Pb may substitute for K^+^ in potassium feldspars and occasionally for Ca^2+^ in minerals. The lead content increases with increasing silica content and, generally, with increasing potassium content, as a result of metasomatic processes that produce K-feldspar; however, feldspar contains most of the lead in the igneous and metamorphic rocks, although there is a general parallelism between the amounts of lead and potassium [36], the correlation matrix of trace elements (Table 7) reveals a slight positive correlation between Pb and SiO_2,_ Cr_2_O_3_ and Al_2_O_3_ contents, as well as a positive correlation with K^+^. Conversely, there is a negative correlation between Pb and MgO, CaO, P_2_O_5_. Fe_2_O_3_, and MnO content. Similarly, Zn, especially at the highest contents of 1590 ppm and 1340 ppm in the Vm_2_ and Vn_2_ samples, is homogeneously distributed throughout most ferromagnesian minerals, commonly following Fe, for which it probably substitutes [9]. Zinc exclusively exists in the divalent state as Zn^+2^ and participates in the formation of a diverse array of minerals. Classified as a compatible element, it shares a nearly similar size or ionic radius with Fe^+2^ and Mg^+2^. Consequently, zinc has a tendency to replace these cations in ferromagnesian minerals. The correlation matrix of trace elements (Table 7) shows no correlation between Zn and Cr_2_O_3,_ TiO_2_ and P_2_O_5_ content, while it exhibits a weak positive correlation with Fe_2_O_3_, a positive correlation with K; conversely, there is a negative correlation with MnO, and MgO, CaO, and Na_2_O content, and weak negative correlation between SiO_2,_ and Al_2_O_3_ content.

An abundance of chalcopyrite and arsenopyrite minerals was also found to be present in R5, with levels of 2030 ppm and 11.2 ppm, respectively, and 5228 ppm and 9 ppm, respectively, in R4. These findings were derived from the high amounts of copper and arsenopyrite. Arsenopyrite and pyrite are two examples of submicroscopic gold-carrying minerals that may be identified by the presence of arsenic, as stated by Chryssoulis and Cabri [8]. The presence of As is the most extensively used index in geochemical exploration [6, 35]. This is because the presence of As is the most reliable indicator of the existence of gold. In addition to iron ore minerals, arsenic may be found in aluminosilicates such as quartz, feldspar, and other varieties. It is likely that the feldspar found in igneous rocks is responsible for at least half of the total arsenic, and there is evidence that arsenic may substitute for silicon, aluminum, and iron in the crystal structure [28], the correlation matrix of trace elements and major oxides shows negative correlation between As and TiO_2_ and Cr_2_O_3_ oxides content, as well as weak negative with SiO_2,_ Al_2_O_3,_ Fe_2_O_3_ and CaO, content, conversely, there is a positive correlation with Na_2_O and MnO content. Sb is clearly a helpful indicator element in the region, with high Sb values of 2.2 ppm in sample Vm1, which indicates that the sulfosalt minerals that are connected with pyrite are likely to be the principal source of Sb in the primary mineralization [25]. There is a possibility that the existence of highly enriched gold mineralizing fluids that have traveled across deeply buried basement structures might be indicated by the enriched barite values that were discovered in the rock samples. Barium is a trace or minor element found in potassium feldspar and mica in common igneous rocks, where it replaces K^+^; granitic rocks often contain slightly more barium than ordinary continental crust material [20], correlation matrix of trace elements shows weak negative correlation between barium and K_2_O content. The rock samples revealed negligible levels of antimony (usually < 2.5 ppm), molybdenum, and silver (< 2 ppm). Only samples Vn1, VF1, Vm1, and Vt1 displayed values of 4.5, 5.1, 4.3, and 4.8 ppm for molybdenum, and only samples Vn1, Vm1, Vf1 and Vb3 had elevated values of silver (18, 9.2, 6.02, and 5.6 ppm, respectively). A few tens of parts per million (ppm) was the highest level of arsenic that was found. Based on the findings of Al-Hwaiti et al. [2], a decrease in the concentration of antimony or arsenic might be an indicator that the top of the hydrothermal system has been stripped away. According to Al-Hwaiti et al. [2], the majority of the samples are characterized by the presence of Fe, which was developed as a consequence of the modification of chalcopyrite and pyrite. Okour et al. [27] and Al-Hwaiti et al. [2] are two examples of researchers who have done investigations in the Wadi Abu Khusheiba region, and their findings are in agreement with these geochemical findings. Oke et al. [26], Ernawati et al. [11], and Stergiou et al. [34] discovered that quartz veins were connected with higher amounts of Pb, As, Sb, Zn, Cu, Ag, as well as rare earth metals. Gold was also shown to be present in these veins.

### Petrography

The findings of the petrographic examination showed that the rock vein samples are felsite rocks that are abundant in quartz. Pyrite and chalcopyrite are the principal ore minerals that are most often found in mineralization veins. The samples, on the other hand, display an alkaline feldspar. On the other hand, hematite and goethite are examples of alteration minerals. These minerals are formed when pyrite and chalcopyrite undergo a certain transformation. These results are in agreement with the geochemical data of major and trace elements; they are in agreement with the work of Al Khawalde [1] on the Aheimir volcanic suite in different parts of southwest Jordan, and they are mostly in agreement with the work of Okour et al. [27] and Al-Hwaiti et al. [2] in the Wadi Abu Khusheiba area.

Factors like as weathering, erosion, and slope impact the physical movement of gold particles or minerals carrying gold from the vein, which is known as the dispersion mechanism. Transport and deposition heterogeneity often results in reduced accuracy and precision. Because of weathering and complexation with organic substances, gold may travel as dissolved ions in surface or groundwater. Because of the more uniform diffusion patterns, this often creates geochemical halos around the vein, which may provide greater precision and accuracy. Environmental variables have a significant impact on the precision and accuracy of gold dispersion mapping. To get trustworthy findings, you need to think about your study's context and choose the right dispersion patterns, analytical approaches, and data processing procedures. Using certified reference materials and blank samples during analysis, conducting repeat analyses of select samples to assess analytical reproducibility, applying appropriate statistical methods to interpret and visualize geochemical data, and validating your interpretation through geological mapping and other methods, this work has achieved enhanced accuracy and precision of your geochemical dispersion.

## Conclusions

- Gold mineralization Source: Major gold mineralization in Wadi Abu Khusheiba is associated with a quartz/silica vein.
- Rock classification: The studied rocks are silica oversaturated, with over 66% SiO2 content and free quartz minerals present.
- Peraluminous characteristics: Based on alumina content, the rock samples exhibit peraluminous properties.
- Rock sample classification: Total alkali silica analysis places all examined rock samples in the rhyolite field.
- Anomalous metal levels: Samples Vn1, Vm1, and Vm5 from the gold vein showed anomalous gold levels. Samples Vn1 and Vm1 also exhibited anomalous silver levels, while sample R3 had an anomalous copper value.
- Correlations among elements: Positive Correlations: Au with Sb, Ag, and Mo, indicating common origins and associations in the area. Negative Correlations: Au with Cu, Pb, Ba, and Zn.
- Indicator metals: Ag, Mo, and Sb are reliable indicators of Au presence.
- Spatial trends: Gold and basic elements show a positive trend in the NW direction within the quartz vein.
- Vein sample analysis: Rock vein samples yielded up to 18 ppm Ag and a maximum of 5 ppm Au.
- The results of petrography revealed that the rock vein samples are quartz-rich felsite rocks. The samples exhibit an alkaline feldspar, and the common primary ore minerals in mineralization veins are pyrite, chalcopyrite and gold, on the other hand, hematite and goethite occur as alteration minerals, resulting from the alteration of pyrite and chalcopyrite
- Contribution to geological understanding: These findings enhance knowledge of the geological history and mineralization processes in Wadi Abu Khusheiba, aiding further exploration and research in this area.

## Data Availability

The data used to support the findings of this study are included within the article.
